# Linking the epigenetic ‘language’ of covalent histone modifications to cancer

**DOI:** 10.1038/sj.bjc.6601575

**Published:** 2004-02-17

**Authors:** S B Hake, A Xiao, C D Allis

**Affiliations:** 1 Laboratory of Chromatin Biology, The Rockefeller University, Box 78, 1230 York Avenue, New York, NY 10021, USA

**Keywords:** histones, chromatin, cancer, epigenetics

## Abstract

Covalent modifications of histones, such as acetylation, methylation, and phosphorylation, and other epigenetic modulations of the chromatin, such as methylation of DNA and ATP-dependent chromatin reorganisation, can play a major part in the multistep process of carcinogenesis, with far-reaching implications for human biology and human health. This review focuses on how aberrant covalent histone modifications may contribute to the development of a variety of human cancers, and discusses the recent findings with regard to potential therapies.

## CHROMATIN REMODELLING

Eukaryotic DNA is intimately associated with a family of small, basic histone proteins, to form a highly ordered and condensed protein : DNA complex termed chromatin. Generally, two different forms of chromatin have been described; heterochromatin is tightly compacted and associated with transcriptionally silent genomic regions, whereas euchromatin has a more open conformation and tends to support transcription (reviewed in Wolffe and Kurumizaka, 1998).

The fundamental unit of the chromatin polymer is the nucleosome, which consists of approximately 147 base pairs of DNA wrapped around an octamer of histone core proteins. This octamer is composed of two copies of each H2A, H2B, H3, and H4, or in some instances, specialised natural variants of these proteins. From this fundamental chromatin unit, N- and, in some cases, C-termini of core histones protrude from this core structure and contact adjacent nucleosomes in a higher-order structure whose details remain elusive (Luger *et al*, 1997). It is now becoming clear that histone-modifying enzymes can alter the structure of these domains and/or influence the binding of ‘effector’ molecules that, in turn, affect patterns of gene expression. Aberrant activity or mis-targeting of these chromatin-modifying activities is proving to have unexpected links to carcinogenesis.

Remodelling of chromatin can be achieved in several different, but interconnected, ways: (1) covalent modification of histones, (2) exchange of ‘generic’ core histones with histone variants, (3) disruption of the basic nucleosome structure and histone DNA contacts, and (4) modification of the DNA itself. As mentioned above, tail domains of histones are subject to a diverse array of covalent modifications that include: lysine acetylation, lysine and arginine methylation, serine and threonine phosphorylation, ADP-ribosylation, ubiquitination, sumolation, and, likely, other yet unknown or poorly appreciated modifications.

Histone acetylation, arguably the best-studied histone modification, occurs at the *ɛ* amino groups of evolutionarily conserved, often invariant, lysine residues most often located in tail domains. Levels of acetylation of the core histones result from the steady-state balance between the opposing activities of histone acetyltransferases (HATs) and histone deacetylases (HDACs). In general, increased levels of histone acetylation (hyperacetylation) are found in more decondensed euchromatin, whereas decreased levels of acetylation (hypoacetylation) are a characteristic of more condensed heterochromatin (Strahl and Allis, 2000; Turner, 2000; Fischle *et al*, 2003b).

**Figure 1:**
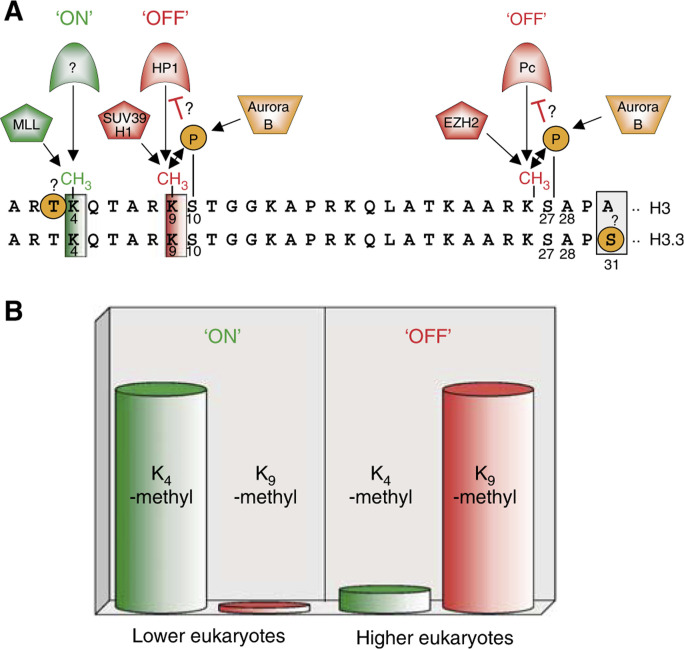
‘Writing’ and ‘reading’ of certain covalent marks in human histone H3 and H3.3 variant. (**A**) Dominant methyl marks are found on lysines 4, 9, and 27 in histone H3, all adjacent to threonine or serine, potential phosphomark carriers (Fischle *et al*, 2003a). Methylation of lysine 4 is generated by the SET domain of MLL, and is connected to gene activation of ceratin target genes (green=‘ON’ mark) (Milne *et al*, 2002). Protein(s) that ‘read’ this mark are not yet identified. Marks that correlate with gene silencing are methylation of lysines 9 and 27, generated by the HMTs SUV39H1 and EZH2, respectively (red=‘OFF’ marks). ‘Readers’ of these repressive marks are HP1 for lysine 9 methylation and Pc for lysine 27 methylation (Fischle *et al*, 2003c). Serines, adjacent to lysines 9 and 27, are shown to be phosphorylated by Aurora B kinase (orange), and might play a role in preventing ‘readers’ from recognising methyl marks. It is not yet known if threonine 3 is also a phospho mark (orange circle). Sequence alignment of the N-termini of H3 with H3.3 variant shows an almost identical sequence, except that alanine 31 in H3 is replaced by serine in H3.3, another potential phospho mark. (**B**) Lower eukaryotes maintain an epigenetic active or permissive state, whereas higher eukaryotes show an epigenetic repressive phenotype. Histone H3 from yeast and *Tetrahymena* are strongly methylated at lysine 4 (‘ON’ mark), but not at lysine 9 (‘OFF’ mark). The opposite was observed for H3 in chicken and humans, where lysine 9 was strongly methylated, but not lysine 4 in H3 (Briggs *et al*, 2001).

Methylation of histones can occur on lysine and arginine residues, giving the cell another layer of regulatory options (in some cases on lysine residues, which are well known to also be acetylation sites; for example, lysine 9 in histone H3). In addition, lysines can be mono-, di-, or tri-methylated, whereas arginines can be mono- or di-methylated (symmetrically or asymmetrically), thus greatly extending the complexity of histone modification-dependent gene regulation. Current evidence suggests that histone arginine methylation is more dynamic, correlating well with gene activation and its loss from target arginines in H3 and H4, with gene inactivation (reviewed in Stallcup, 2001; Bannister *et al*, 2002; Davie and Dent, 2002). In contrast, lysine methylation appears to be a more stable mark, with what appears to be a more complicated readout (reviewed in Zhang and Reinberg, 2001; Lachner, 2002). For instance, as depicted in Figure 1A

, methylation of lysine 4 in histone H3 correlates with gene activation, whereas methylation of lysines 9 and 27 in histone H3 correlates with repression (reviewed in Bannister *et al*, 2002; Fischle *et al*, 2003b). These marks are ‘written’ by histone methyltransferases (HMTs), many of which contain a conserved SET (Su(var)3–9, Enhancer-of-zeste, Trithorax) domain (reviewed in Schneider *et al*, 2002). Insights into SET domain structure and mode of catalysis are beginning to emerge (Min *et al*, 2002). ‘ON’ and ‘OFF’ methyl marks are ‘read’ by chromodomain-containing proteins, such as heterochromatin protein 1 (HP1) and Polycomb (Pc) (Figure 1A), and it is becoming clear that these proteins specifically recognise methyl marks, depending on their location in the histones (Fischle *et al*, 2003c).

Phosphorylation is another important and long-appreciated histone modification that is often associated with chromosome/chromatin condensation that includes mitosis, meiosis, apoptosis and DNA damage, events regulated by different histone kinases (for example, members of the Aurora/AIK family; reviewed in Fischle *et al*, 2003a). Histone phosphorylation is also closely correlated with chromosome decondensation events (such as the immediate-early response to mitogens), suggesting a ‘split personality’ for certain modifications that remains to be fully understood (for example, at serine 10 in H3) (Cheung *et al*, 2000).

The histone ‘code’ hypothesis (Strahl and Allis, 2000; Fischle *et al*, 2003b) has been put forward that may explain the seemingly complex nature of reported patterns of histone modification readouts. Formally, this hypothesis states that one modification or specific combinations of histone modifications can affect distinct downstream events by altering the structure of the chromatin and/or generating a binding platform for protein effectors that can specifically recognise the modification(s) and initiate gene transcription or repression. Alternative views for how distinct patterns of histone modifications may coordinate distinct biological readouts have also been expressed (Schreiber and Bernstein, 2002; Kurdistani and Grunstein, 2003).

The finding that cells contain alternative, more specialised versions of the four canonical core histones is well documented. Numerous histone variants have been described in all eukaryotes for all histone classes except histone H4. While poorly appreciated and poorly understood, histone variants afford the cell a chance to alter the primary sequences contained within the nucleosome. Active replacement of histone variants into ‘normal’ nucleosomes may, in turn, alter the regulatory options available, including choices of post-translational modifications. For example, in several cases, replacement of serine for alanine distinguishes the H3 histone from that of a H3 variant, H3.3 (Figure 1A). These differences suggest alterations in phosphorylation that may reflect functional differences between the two histones (Ahmad and Henikoff, 2002). In keeping, a specialised H2A variant, H2A.X, has recently been shown to act as if it is a ‘caretaker’ of our genome upon DNA damage (Figure 4B, C; Bassing *et al*, 2003; Celeste *et al*, 2003). As histone variants also are enriched in other critical regions of the genome, such as centromers and inactive X chromosome (reviewed in van Leeuwen and Gottschling, 2003), it seems likely that histone variants themselves hold many more secrets.

ATP-dependent chromatin remodelling multi-protein complexes, such as SWI/SNF, noncovalently alter or disrupt the nucleosome structure, promoting transient loosening of DNA–histone contacts and facilitating the binding of transcription factors (Muchardt and Yaniv, 1999). Emerging evidence suggests that some of these ATP-dependent remodellers may function to shuffle histone variants into and out of chromatin (Figure 4A) (Mizuguchi *et al*, 2003).

**Figure 2:**
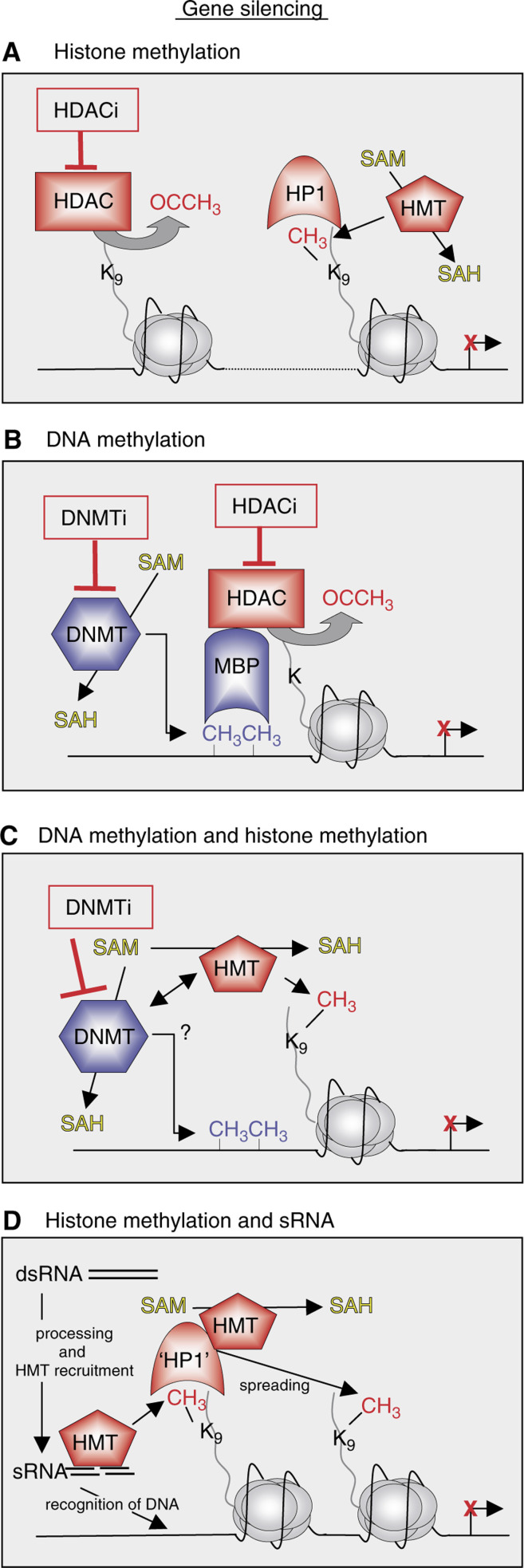
Epigenetic modifications leading to gene silencing. (**A**) Gene repression through histone methylation. Histone deacetylase deacetylates lysine 9 in H3, which can then be methylated by HMTs. Methylated lysine 9 in H3 is recognised by HP1, resulting in maintenance of gene silencing. (**B**) Gene repression involving DNA methylation. DNA methyltransferases methylate DNA by converting SAM to SAH, a mechanism that can be inhibited by DNMT inhibitors (DNMTi). MBPs recognise methylated DNA and recruit HDACs, which deacetylate lysines in the histone tails, leading to a repressive state. (**C**) Interplay between DNMTs and HMTs results in methylation of DNA and lysine 9 in H3, and consequent local heterochromatin formation. The exact mechanism of this cooperation is still poorly understood. (**D**) Specific gene repression by small RNAs (sRNAs). Transcription of repetitive DNA sequences lead to double-strand RNA (dsRNA) generation by still poorly understood mechanisms, and dsRNA is later processed to sRNAs. sRNAs associate with and recruit HMTs to the complementary DNA sequence, where HMTs locally methylate lysine 9 in H3. Methylated lysine 9 is recognised by ‘HP1’ that forms a complex with HMTs to spread the repressive mark to other histones, until reaching a boundary. ‘Writers’ and ‘readers’ of DNA epigenetic marks are shown in blue, and proteins involved with repressive histone marks are depicted in red. Tail length has been exaggerated for clarity.

Outside the covalently marking histone proteins in localised genomic locations, such as hyperacetylating specific promoter elements or other such upstream activating sequences, it appears that other long-range ‘indexing’ systems also operate to mark the epigenome. For example, using chromatin immunoprecipitation (ChIP) assays and site-specific methylation antibodies, elegant studies have shown that lysine 4 methylation *vs* lysine 9 methylation in histone H3 can mark relatively large chromosomal domains (about 25–50 kb) with ‘active’ *vs* ‘inactive’ signatures often interrupted by sharp transitions or boundaries that are poorly understood (reviewed in Jenuwein and Allis, 2001; Zhang and Reinberg, 2001; Felsenfeld and Groudine, 2003). Moreover, as depicted in Figure 1B, it appears that the repressive lysine 9 methylation mark in histone H3 is a dominant histone mark in mammalian histones (‘OFF’), whereas in lower eukaryotes methylation of lysine 4 in H3 (‘ON’) dominates (Briggs *et al*, 2001). This suggests that epigenetic mechanisms exist that lead to a silencing default or ground state in higher organisms. Figure 2

shows some examples of how the cell may achieve gene silencing using epigenetic-based mechanisms. One possibility to repress gene transcription, as shown in Figure 2A, is the removal of acetyl groups from H3 (activation mark) by HDACs, a process that can be inhibited by HDAC inhibitors (HDACi). Unmodified lysine 9 is in turn methylated by HMTs by converting *S*-adenosylmethionine (SAM) to *S*-adenosylhomocysteine (SAH). This metabolism can be directly influenced by dietary take-up, and aberrant SAM/SAH ratio can affect health and potentially contribute to carcinogenesis (Huang, 2002). HP1 recognises this ‘OFF’ mark and maintains gene silencing.

Not only do histone modifications influence chromatin structure, but modifications of the DNA itself can also lead to remodelling of chromatin and consequently result in gene silencing. DNA methylation results from the activity of a family of DNA methyltransferase (DNMT) enzymes, which catalyse the addition of a methyl group to cytosine residues at CpG (adjacent cytosine and guanine nucleotides) islands, also by converting SAM to SAH (see Figure 2B). A family of proteins with a methyl-binding domain (MBD) can recognise methylated DNA, and have been shown to associate with large protein complexes containing HDACs and chromatin-remodelling activities. As a result, histones are deacetylated and gene transcription is most often repressed. It has also been suggested that DNA methylation could lead to gene silencing by MBD proteins that recruit HMTs, which methylate lysine 9 in histone H3 and subsequently repress gene transcription (Figure 2C) (reviewed in Rice and Allis, 2001; Brown and Strathdee, 2002).

More recently, genetic links between histone methylation, notably tri-methylation at lysine 9 in histone H3, and DNA methylation have appeared in organisms as diverse as fungi, plants, and mice (Hashimshony *et al*, 2003; Lehnertz *et al*, 2003). Thus, histone methylation and deacetylation and DNA methylation have direct and indirect links that connect each of them to each other and to the readout of higher-order chromatin structures. The breakthrough findings that histone methylation, notably lysine 9 methylation in histone H3, may also be ‘guided’ by small heterochromatin-associated RNAs by a mechanism that remain unclear (see Figure 2D) suggest that the cell has invested considerable energy in covalently marking what is now referred to as the ‘epigenome’ (reviewed in Grewal and Moazed, 2003). We and others favour the view that histone deacetylation, histone methylation, DNA methylation and the production of small RNAs, all work together in various orders to bring about an efficient silent state (Grewal and Moazed, 2003). Remarkably, these epigenetic silencing mechanisms can be targeted to ectopic genes, thereby marking chromatin in such a way that this silencing state is inherited over many cell generations (Snowden *et al*, 2002; Ayyanathan *et al*, 2003).

**Figure 3:**
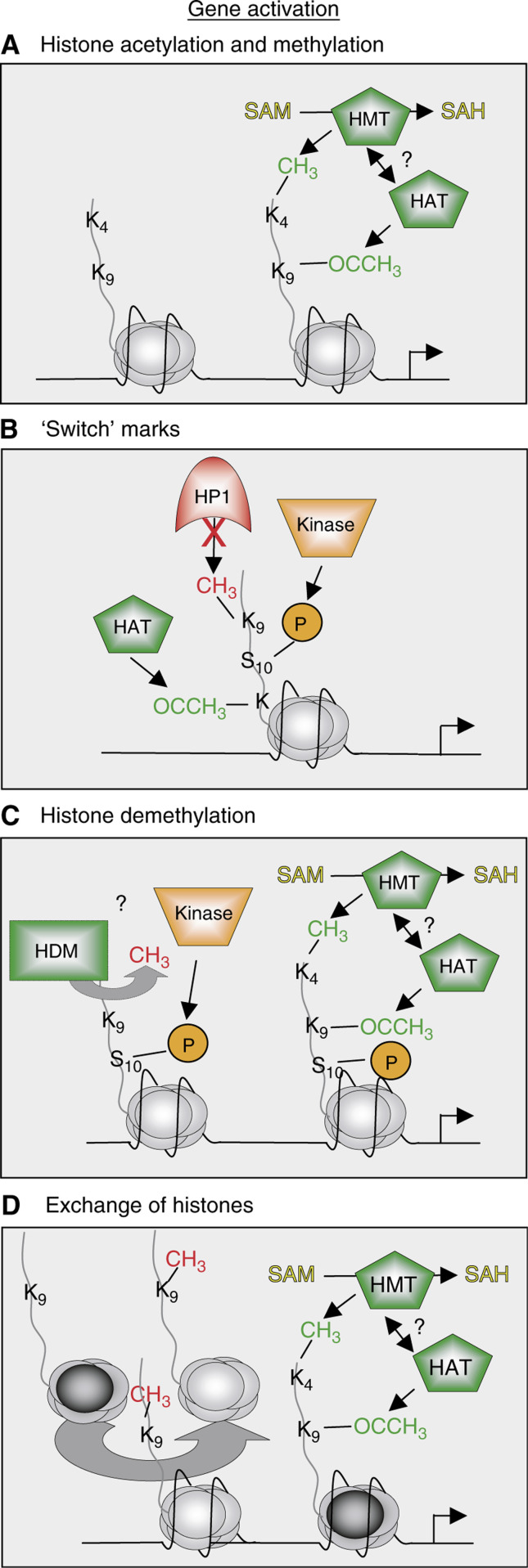
Epigenetic modifications leading to gene activation. (**A**) Setting ‘ON’ marks in histone H3 to activate gene transcription. Lysine 4 in H3 is methylated by HMT (for example MLL) and lysine 9 is acetylated by HAT, allowing genes to be transcribed. It is not known, if HMTs and HATs have a direct connection to each other. (**B**) In the postulated ‘switch’ hypothesis (Fischle *et al*, 2003a), phosphorylation of serines or threonines adjacent to lysines displaces histone methyl-binding proteins, accomplishing a binding platform for other proteins with different enzymatic activities. For example, phosphorylation of serine 10 in H3 may prevent HP1 from binding to the methyl mark on lysine 9. Other lysines in H3 may be acetylated by HATs, therefore overwriting the repressive lysine 9 methyl mark and allowing activation. (**C**) Although there is no HDM identified to date, one can speculate that, if this enzyme exists, serine 10 phosphorylation in H3, for example, by Aurora kinases, can lead to recruitment of HDMs that in turn demethylate lysine 9 in H3. Histone acetyltransferases might then acetylate lysine 9 and HMTs methylate lysine 4, resulting in the loosening of the chromatin structure and allowing gene transcription. (**D**) Repressive-marked histones are exchanged with unmodified (or active) counter parts (dark circles) that are then acetylated at lysine 9 by HATs and methylated at lysine 4 in H3 by HMTs, for example, MLL, leading to gene activation. Proteins involved with repressive histone marks are depicted in red, ‘writers’ and ‘readers’ of histone activation marks are shown in green, kinases are orange and phospho marks are depicted as orange circles.

On the other hand, epigenetic mechanisms have also evolved to ensure that specific genes are not only silenced, but also activated at appropriate times during the cell cycle or during development. Figure 3

shows some examples of the coordinated interplay between kinases, HATs and HMTs marking the chromatin in such ways that genes are transcribed. As shown in Figure 3A, the epigenetic ‘language’ of gene activation contains ‘ON’-marks like lysine 4 methylation and histone hyperacetylation. But how cells manage to switch from repressive chromatin that contains, for example, lysine 9 methylation in histone H3, to an active state with nucleosomal ‘ON’ marks is not known. A new theory, the so-called binary ‘switch hypothesis’ is based on the observation that the best studied methyl marks in the H3 tail (lysine 4, 9, and 27) are all adjacent to serine or threonine (potential phospho marks), and discusses the arising regulatory possibilities with regard to gene repression and activation (see Figure 1A; Fischle *et al*, 2003a). This concept proposes that phospho marks permit the docking of methylmark ‘readers’, allowing gene activation by at least two possible mechanisms. In the first scenario, see Figure 3B, phosphorylation of serine 10 in H3 interferes with the blading of HP1 to the methyl mark on lysine 9, and lead to an only temporary switch from repression to activation by additional histone acetylation. This process can be converted back through a more dynamic and reversible mechanism, since phospho marks can be removed easily. To switch from a repressive to a stable active state, another possibility can be envisaged. As depicted in Figure 3C, a not yet identified histone demethylase (HDM) may recognise the phospho mark on serine 10 and erase the repressive methylmark from lysine 9, thereby allowing HATs to acetylate lysine 9, producing an activation -mark. Alternatively, as depicted in Figure 3D, repressive-marked histones may be exchanged with newly synthesised histones or histone variants that can be modified with activation marks, such as methylation of lysine 4 and histone hyperacetylation.

The pathways depicted in Figures 2 and 3 are proposed to take place in normal cells, leading to appropriate gene silencing and activation. It remains unclear to what extent these pathways go awry in the progression of human neoplasia. However, as none of these pathways shown involve DNA mutation, understanding the fundamental mechanisms of epigenetic silencing promises to afford opportunities to de-silence (or de-activate) genes that may have been inappropriately silenced (or activated) during transformation and tumour progression. Below, we highlight some of the more clear examples where enzymes that ‘write’, or protein effectors that ‘read’ the covalent language of histone modifications, have been linked to human cancer. Exciting recent developments in this field are beginning to surface and show remarkable promise in clinical trials, as epigenetic forms of dysfunctional pathways are uncovered in human cancer and experimental therapeutic strategies are advanced. Our review intends to stay centred on epigenetic-based mechanisms, pathways, and players, many of which are superficially depicted in all figures. Interested readers are encouraged to refer to other excellent reviews on emerging chromatin links to human biology and disease, notably cancer (Chakraborty *et al*, 2001; Klochendler-Yeivin and Yaniv, 2001; Beaudet, 2002).

## CANCER AND EPIGENETICS

Epigenetic-based mechanisms that lead to carcinogenesis can be divided into at least three different categories: (1) repression of normally active genes, for example tumour-suppressor genes, generated by the single or combined activities of HDACs, HMTs, DNMTs, and SWI/SNF, (2) activation of normally silent genes, for example, oncogenes, where HAT and HMT activities, and SWI/SNF proteins are involved, and (3) replacement of core histones with specifically modified histone variants.

### Aberrant gene repression and cancer

As depicted in Figure 2, the interplay of different histone modification and DNA methylation enzymes leads to transcriptional gene repression. Deregulation of this cooperation or mis-targeting of these enzymes can lead to neoplasia, and a few examples will be discussed below.

#### Acute myelogenous leukaemia (AML)

Local histone deacetylation is generated by the activity of HDACs and results in silencing of genes (see Figures 2A, B). Deregulation of HDAC activity and resulting histone deacetylation at normally active sites can lead to cancer.

The acute myeloid leukaemia gene 1 (RUNX1/AML1/CBFA2) is one of the most frequent targets for chromosomal translocations in leukaemia. The AML1 protein binds to the co-repressors Groucho/transducin-like enhancer (TLE) and Sin3, and also the HAT p300/CBP complex (Javed *et al*, 2000), and guides their activity to AML target genes. Therefore, depending on the specific target gene, AML1 is capable of functioning as both a transcriptional activator and repressor.

One common oncogenic event is the t(8;21) translocation that fuses the DNA-binding domain of AML1 with ETO (MTG8). ETO, the mammalian homologue of *Drosophila* Nervy, is a nuclear phosphoprotein that is expressed in haematopoietic progenitors. ETO interacts with multiple HDACs and Sin3, N-CoR, and SMRT co-repressors. The AML1–ETO fusion protein localises to AML1 target genes, where it, in contrast to the wild-type AML1 protein, actively suppresses transcription via the co-repressors N-CoR/Sin3/HDAC1, directly. Since AML1 is required for differentiation of haematopoietic cells, AML1–ETO expression leads to a block in myeloid development and leukaemic transformation (reviewed in Jones and Saha, 2002). Interestingly, point mutations in AML1 resulting in haploinsufficiency are associated with familial thrombocytopenia and a markedly increased risk for the development of acute myeloid leukaemia (Song *et al*, 1999).

#### RB

An excellent example of the cooperate interplay of HDACs, HMTs, and SWI/SNF (see Figure 2A) in carcinogenesis is the deregulation of the tumour suppressor retinoblastoma protein (Rb) pathway, which is mutated in a majority (90%) of human solid tumours. Rb pathway defects include gain-of-function mutations of the cyclin-dependent kinase cdk4/cyclin D, which activates Rb through phosphorylation, and loss-of-function mutations of p16 and Rb. Rb regulates cell proliferation by controlling a set of transcription factors (the E2F family of proteins) that activate genes involved in the G1/S cell cycle transition. Hypophosphorylated Rb inactivates E2F in early G1 phase and becomes hyperphosphorylated during G1/S transition, resulting in the release and activation of E2F (Hanahan and Weinberg, 2000).

At least two mechanisms contribute to the inactivation of E2F. Firstly, Rb binds to the transactivation domain of E2F and subsequently prevents E2F from association with promoter regions, resulting in the silencing of its target genes. Secondly, Rb can be targeted to specifc sites through association with E2F, thereby actively repressing transcription by recruiting HDAC1 and SWI/SNF. Histone deacetylase 1 binding to the Rb–E2F complex leads to the transcriptional silencing of cell cycle-related genes, including cyclin E. BRG1/BRM, a component of SWI/SNF, is also found to be associated with Rb, and this complex cooperatively induces G1/S cell cycle arrest. The Rb–BRG1/BRM complex seems to be required for the repression of cyclin A and cdk2 genes, which are important for G2/M phase transition, but not cyclin E, which is necessary for S-phase entry. This suggests that distinct histone remodelling and modification complexes may be required for Rb to repress different target genes (reviewed in Harbour and Dean, 2000). Notably, loss of integrase interactor 1 (INI1), a core component of the SWI/SNF complex, is a characteristic feature of some human tumours including rhabdoid and primitive CNS tumours (Sevenet *et al*, 1999). In addition, mutations in Brg-1 have been identified in a variety of human tumours (reviewed in Muchardt and Yaniv, 2001). Rb has been shown to interact with the human homologue of Polycomb (PcG) HPC2, and the HMT *Suv39H1*, both of which seem to play a role in transcriptional gene repression by methylation of lysine 9 and/or 27 in histone H3 (Dahiya *et al*, 2001; Nielsen *et al*, 2001). These data suggest an important role for Rb family proteins in linking sequence-specific transcription factors with a variety of histone remodelling and modification enzymes, an observation that may also explain why Rb is a favourite target for mutations in cancer.

#### EZH2

Mammalian PcG proteins are HMTs, and can repress transcription by at least two different mechanisms. (1) The PcG complex that contains EED and enhancer of zeste homolog (EZH) proteins 1 and 2 recruits HDACs and represses transcription of target genes by the combinatory action of local histone methylation and deacetylation. (2) The Polycomb repressive complex 1 (PRC-1) contains polycomb 2 (HPC2), polyhomeotic (HPH), BM1 and Ring-finger protein 1 (RING1) proteins and associates with the SWI/SNF chromatin remodelling complex, and negatively regulates chromatin accessibility (Shao *et al*, 1999).

The Drosophila E(Z) protein is shown to methylate lysines 9 and 27 in H3, and causes stable changes in the chromatin, leading to the repression of target genes (Su *et al*, 2003). Overexpression of the human E(Z) homologue EZH2 has been observed in prostate cancer and lymphomas, and is linked to increased cell proliferation (Sellers and Loda, 2002; Varambally *et al*, 2002). Although EZH2 target genes have not been directly linked to cancer development, evidence for EZH2 function in at least B-cell development is beginning to emerge. Mice deficient for EZH2 showed impaired early B-cell development and decreased rearrangement of the immunoglobulin heavy chain (IgH). Since EZH2 has been shown in this study to specifically methylate lysine 27 in H3, it is plausible that EZH2 absence reduces both basal and interleukin-7-induced histone H3 lysine methylation, although the exact mechanism(s) remains elusive (Su *et al*, 2003). Since EZH2 plays a role in B-cell development in mice, it will be interesting to see, if human lymphomas show an increased methylation of lysine 27 (and possible lysine 9) in histone H3 at EZH2 target genes. And, if so, is the resulting repression of these genes oncogenic?

#### DNA methylation

As described above, DNA methylation is an additional mechanism to silence genes (see Figures 2B, C), and considerable work has been invested to study the connection between aberrant DNA methylation and cancer.

At the early stage of neoplasia, cells develop genome-wide hypomethylation of DNA. Later on, promoter regions of several tumour-suppressor genes, such as p16 and BRCA1, are hypermethylated, leading to downregulation of transcription. Numerous pathways are affected by aberrant DNA hypermethylation, including cell cycle, DNA repair, and hormonal responses, and therefore alter the perfect epigenetic cell equilibrium. The mechanisms leading to this oncogenic profile of hypermethylation and different numerous tumours are currently not understood (Bachman *et al*, 2003).

The other remarkable characteristic of neoplasias is the early occurrence of a global genomic hypomethylation. This loss of DNA methyl marks is achieved mainly by hypomethylation of exons and introns of gene-rich regions and repetitive DNA sequences. This phenomena is also not well understood, but may contribute to carcinogenesis by several mechanisms, such as loss of imprinting, chromosomal instability, and reactivation of transposable elements (reviewed in Esteller, 2003). Due to limited space, we are not able to give a more detailed overview about this topic, and we encourage interested readers to refer to other excellent reviews on the connection of DNA methylation and cancer (Dunn, 2003; Jaenisch and Bird, 2003; Laird, 2003; Szyf, 2003).

### Aberrant gene activation and cancer

Not only does the repression of genes by the activity of HDACs, HMTs, DNMTs, and SWI/SNF have important implications in neoplasia, the transcriptional activation of genes at unusual times in cell differentiation and development by the activities of kinases, HMTs, HATs, and SWI/SNF (see Figure 3) can also lead to cancer. One excellent example is MLL, which will be discussed below.

#### MLL

Lysine 4 methylation in histone H3 is often regarded as an ‘activating’ epigenetic mark that is abundant in lower eukaryotes, but only sparsely presented in mammals (see Figure 1B) (Briggs *et al*, 2001). Interestingly in yeast, Set1 is solely responsible for introducing lysine 4 methylation in H3. The Set1 SET domain mostly resembles the *Drosophila* ‘positive’ memory protein trithorax, and the mixed-lineage leukaemia (MLL/HRX/acute lymphocytic leukaemia (ALL)-1) SET domain in humans.

In cells with normal amounts of wild-type MLL protein, the overall MLL activity appears to be dependent on the balance between activation and repression. On the one hand, MLL has been reported to repress transcription by recruiting PcG proteins and/or HDAC1 (Xia *et al*, 2003). On the other hand, MLL's SET domain can generate the ‘ON’ methyl mark on lysine 4 in H3 (Milne *et al*, 2002) and associates with INI1 (Rozenblatt-Rosen *et al*, 1998), and, additionally, MLL is shown to interact with the HAT CBP (Ernst *et al*, 2002). These partners and intrinsic activities lead to an open chromatin structure and transcriptional activation of MLL target genes in early development.

One of the best understood targets of MLL are the clustered homeobox (Hox) genes, which play important roles in axial morphogenesis, patterning, and haematopoetic differentiation (Krumlauf, 1994). Individual Hox gene expression is high in progenitor cells, and is downregulated during differentiation and maturation. Expression of Hox genes (e.g. Hoxa9) is upregulated in human leukaemias carrying MLL rearrangements, and it has been shown recently that transformation by MLL fusion proteins requires Hoxa7 and Hoxa9 (Ayton and Cleary, 2003), indicating that these are pivotal for MLL-associated leukaemogenesis.

The MLL gene is involved in many chromosomal translocations associated with ALL and AML (reviewed in Ayton and Cleary, 2001). In case of leukaemogenic MLL fusions, the balance of MLL's repression and activation functions might be disturbed. Rearrangements of MLL that occur in leukaemia consistently delete the C-terminus, containing plant homeodomain (PHD) fingers, CBP-binding domain, and SET domain, and replace these sequences with one of over 40 different translocation partners that in general share little sequence homology. Since MLL fusion proteins lack the SET domain to methylate lysine 4 in H3 (‘ON’ mark) and CBP-binding domain, it is unclear how MLL fusions lead to Hox gene overexpression and subsequently to leukaemia. Interestingly, all MLL fusion proteins also delete the PHD finger domain, which binds to Cyp33, a cyclophilin family member (Fair *et al*, 2001). In wild-type cells, Cyp33 binding to MLL increases the association of HDAC1 to MLLs repression domain. In the context of MLL fusion proteins, HDAC binding to the repression domain may not be as strong, due to the lack of PHD finger domain and loss of Cyp33 interaction. This may also affect the binding of PcG proteins, ultimately altering the balanced function of the protein, leading to the activation of normally transcriptional silent genes.

Another way to explain the altered activity of rearranged MLL might be found in the different MLL fusion partners and their biological functions, because some play a role in chromatin remodelling processes themselves. Fusions of MLL with, for example, CBP, t(11;16)(q23;p13.3), retain the HAT domain of CBP, and might lead to leukaemia by promoting histone acetylation of genomic regions targeted by MLL, and allowing transcriptional activation (Sobulo *et al*, 1997). Another fusion partner of MLL is ENL, a subunit of the SWI/SNF complex. MLL–ENL fusion proteins, t(11;19), associate and cooperate with SWI/SNF complexes to activate transcription of the Hoxa7 promoter (Nie *et al*, 2003). Fusion of MLL to AF10, t(10;11)(p12;23), creates a protein that could interact with GAS41, a member of the basal transcription factor complexes TFIID and TFIIF, and an interacting component of SWI/SNF, and potentially activate Hox gene expression by aberrant chromatin remodelling (Debernardi *et al*, 2002). Intriguingly, dimerisation of the truncation MLL molecules appears to be an alternate mechanism of MLL oncogenesis that also results in Hox gene upregulation (Martin *et al*, 2003; So *et al*, 2003). Collectively, these data suggest that MLL can activate genes at inappropriate times by mis-targeting histone mark ‘writers’, consequently modifying the chromatin to allow gene activation.

### Histone variants and cancer

**Figure 4:**
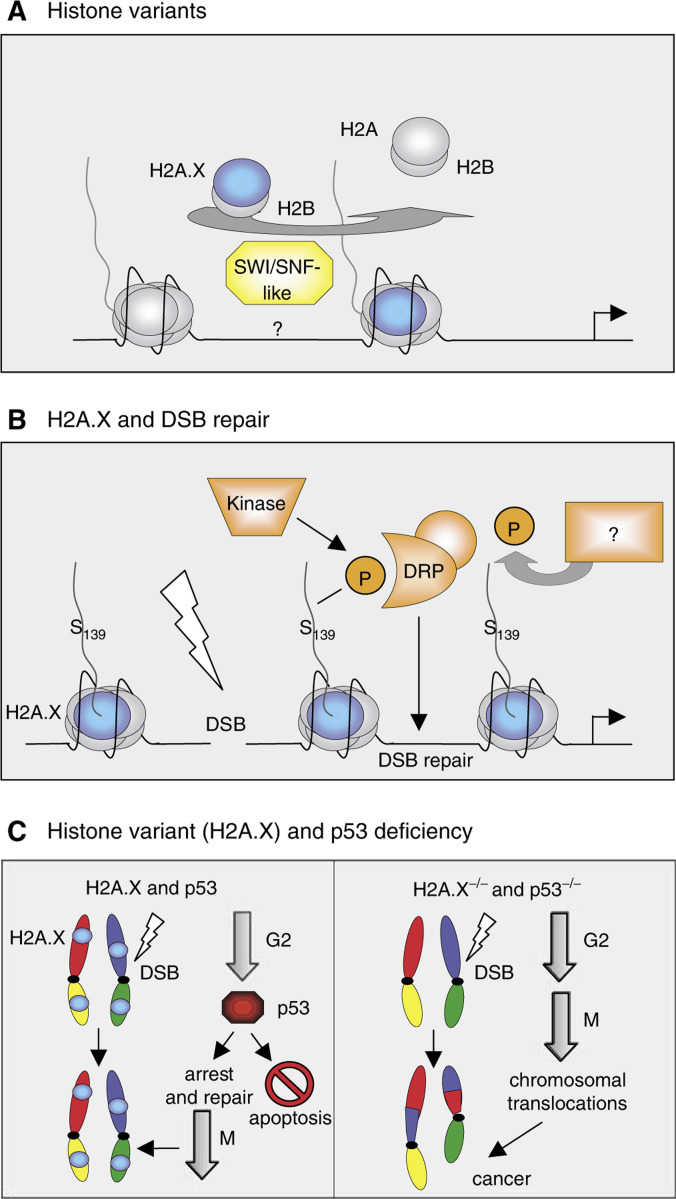
Histone variants and their importance in diverse biological pathways. (**A**) Histone variants, such as H3.3, shown in Figure 1A, are integrated into nucleosomes by a yet not understood mechanism. New data suggest that an ATP-dependent chromatin remodelling complex related to SWI/SNF is involved in the replacement of histones with their variant counterparts (shown here is the exchange of H2A with the variant H2A.X (depicted in blue)). (**B**) Importance of the histone variant H2A.X (depicted in blue) in DNA damage repair. Upon DSB, serine 139 in H2A.X is phosphorylated by phosphatidylinositol 3-kinase, a mark that is recognised by a complex of DNA repair proteins (DRP) that restore the structure of the DNA. After successful DSB repair, serine 139 is dephosphorylated by a yet unknown phosphatase. (**C**) Deficiency of histone variant H2A.X (blue circle) has critical implications for genomic stability. As shown in Figure 4B, H2A.X is important in DNA damage repair and needs p53 to arrest the cell to allow DSB repair. Alternatively, if the DNA damage is severe, p53 activates the apoptosis pathway, preventing thereby mutations and/or chromosome translocations (left site). Loss of both H2A.X and p53 can lead to chromosomal rearrangements after DSB and result in cancer (right site).

Many efforts have been invested to study the products and functions of chromosomal rearrangements and translocations in leukaemia and lymphoma. Mammalian cells, in general, are engineered to keep the genome stable, but little is known about how cells achieve their daunting task to prevent oncogenic events. Not only are proteins with enzymatic activity that covalently modify histones or DNA important, but the identity of the histones themselves might also play an important role in gene regulation. One excellent example of this is provided with the H2A histone variant H2A.X. Recent studies show that DNA damage-induced phosphorylation of H2A.X may be critical to maintain genomic stability. Thus, in addition to alter the gene expression profile without changing the primary DNA sequence, epigenetic factors also regulate genomic integrity (see Figure 4

).

#### H2A.X

Six minor variants of human H2A histones have been reported to date. H2A.X, which represents roughly 10–15% of total H2As in human chromatin, is incorporated into the genome seemingly randomly, and has a longer C-terminus than all other H2A species. Many studies demonstrated that serine 139 of the SQEY motif in the C-terminus is hyperphosphorylated in response to DNA double-strand breaks (DSBs) upon DNA damage, and during meiotic recombination, DNA replication, and V(D)J recombination at the T-cell receptor and immunoglobulin loci. As shown in Figure 4B, H2A.X is phosphorylated in nucleosomes adjacent to DSBs and protein factors involved in DNA repair and signalling pathways are recruited to this site (Paull *et al*, 2000; Redon *et al*, 2002).

Despite the observation that H2A.X-deficient mice have increased genomic instability, they are not prone to tumour development (Celeste *et al*, 2002). But when crossed onto p53-deficient backgrounds, H2A.X deficiency accelerates the lymphocytic tumour development observed in p53-deficient mice (Bassing *et al*, 2003; Celeste *et al*, 2003). As depicted in Figure 4C, this acceleration is correlated with an increased translocation rate in these tumours, but the contributing molecular mechanism(s) remains to be identified. H2A.X seems to function as a genomic ‘caretaker’ by helping other factors, such as p53, to prevent erroneous repair of damaged DNA (reviewed in Downs and Jackson, 2003). Interestingly, the genomic locus for human H2A.X, 11q23.3, has been mapped to a region that is frequently altered in human cancers, possibly implicating similar functions in humans (Monni and Knuutila, 2001).

## CONCLUSION AND PERSPECTIVE

Epigenetics can be defined as heritable changes in gene expression that operate outside of changes (for example, mutations) in DNA itself. Collective studies, reviewed in part here, underscore the fact that all transcription-based regulatory phenomena must take place within a chromatin infrastructure; chromatin is the physiological template of the genome. Emerging evidence now indicates that cell cycle progression, DNA replication, DNA repair, programmed DNA rearrangements, imprinting phenomena, germ-line silencing, developmentally coordinated stem cell divisions, and chromosome stability and identity are all influenced by such epigenetic alterations of chromatin structure. These discoveries have revealed a fundamental and critical regulatory system beyond the sequence information of our genetic code that is maintained in histone proteins as major carriers of epigenetic information (Felsenfeld and Groudine, 2003). Based on the recent progress, it seems likely, if not certain, that epigenetics, in part dictated through a covalent ‘language’ operating in the histone proteins, will touch upon all aspects of biology with far-reaching implications for human biology and disease, notably cancer.

In support, emerging evidence lend support to an emerging view that aberrant chromatin remodelling events play an important role in carcinogenesis; a growing number of HDAC and DNMT inhibitors (HDACi and DNMTi, respectively) are being developed for cancer treatment (see Figure 2). Suberoylanilide hydroxamic acid (SAHA), to name only one HDACi, is currently in clinical trials for a variety of solid tumours and haematologic malignancies, and shows promising results, although the exact mechanism(s) and targets of its anticancer function remain unclear.

Many tumours are found to have aberrantly hypermethylated CpG islands, a finding that is applicable for the detection of a wide range of tumour types. Promoter hypermethylation of CpG islands in tumour-suppressor genes, for example, BRCA1, occurs frequently in tumorigenesis. Tumour treatment with DNMTis, such as 5-aza-2′-deoxycytidine, combined with HDACis has achieved moderate success in several patients with acute promyelocytic leukaemia (Lo Coco *et al*, 2002).

Finally, it seems likely, if not certain, that aberrant phosphorylation and ADP-ribosylation of histones also play a role in tumorigenesis, since Aurora B kinase has been shown to phosphorylate serines in H3 (Figure 1A) and act as an oncogene in human cancer and mice studies (reviewed in Balmain *et al*, 2003). Additionally, increased ADP-ribosylation of histones and nonhistone chromosomal proteins has been observed in oral tumours (Das, 1993). Also, while most of the above studies have focused attention on the major core histones, emerging evidence suggests that histone variants themselves will hold critical epigenetic information that distinguish it from what is carried by the major histones. H2A.X, for example, is a dosage-sensitive suppressor of oncogenic translocations and tumours in mice (Bassing *et al*, 2003; Celeste *et al*, 2003), and, remarkably, this histone variant is phosphorylated at a unique serine in its C-tail (Ser139) in a pathway induced by DSB (see Figure 4B). The H3-like variant CENP-A is specifically localised in centromeres, and phosphorylation of serine 7 (Zeitlin *et al*, 2001) is implicated in mitosis. A recent study showed that overexpression of CENP-A leads to mis-targeting and is found in colorectal cancer tissues (Tomonaga *et al*, 2003). Thus, a more generalised concept is emerging that histone proteins, and their post-translational ‘fingerprints’ may well have direct links to human cancer.

Despite remarkable progress in this area, we are just beginning to scratch the secrets of the histone ‘language’, and many more mysteries remain to be solved. How do cells memorise the epigenetic ‘code’ over many cell generations? And how to read and understand this complex ‘code’, that is so different from the genetic code, so that predictions can be made in the future about the fate of a cell in specific situations. Taking one step further back, we and others are beginning to favour the view that medical genetics may well be neglecting epigenetics in other diseases with complex and poorly understood origins that seem to not follow the classical rules of Mendelian genetics (Beaudet, 2002). As we turn the corner of the 50th birthday of the Watson/Crick DNA double helix, we look forward to gaining insights into heritable mechanisms that operate outside of the DNA. In the epigenetic mechanisms described here, the DNA remains essentially unaltered or ‘wild-type’ in sequence. Thus, understanding how to manipulate its expression (i.e. to de-silence tumour suppressors or to inactivate oncogenes) in an epigenetic/chromatin context promises to lead to exciting advances in our understanding of normal development as well as pathological abnormalities leading to neoplasia.
